# Feto-placental endothelial dysfunction in Gestational Diabetes Mellitus under dietary or insulin therapy

**DOI:** 10.1186/s12902-023-01305-6

**Published:** 2023-02-23

**Authors:** Jing-Jing Wang, Xi Wang, Qian Li, Hua Huang, Qiao-Ling Zheng, Qin Yao, Jun Zhang

**Affiliations:** Department of Clinical pharmacy, First Affiliated hospital of Kunming Medical University, Yunnan, China

**Keywords:** Gestational Diabetes Mellitus, Feto-placental, Endothelial dysfunction, Glucose transporters

## Abstract

**Objective:**

Gestational diabetes mellitus (GDM) is a serious complication in pregnancy. Despite controlling the plasma glucose levels with dietary intervention (GDM-D) or insulin therapy (GDM-I), children born of diabetic mothers suffer more long-term complications from childhood to early adulthood. Placental circulation and nutrient exchange play a vital role in fetal development. Additionally, placental endothelial function is an indicator of vascular health, and plays an important role in maintaining placental circulation for nutrient exchange. This study was conducted to assess changes in fetal endothelial dysfunction in GDM under different interventions during pregnancy.

**Methods:**

The primary human umbilical vein endothelial cells (HUVECs) were obtained from normal pregnant women (n = 11), GDM-D (n = 14), and GDM-I (n = 12) patients. LC-MS/MS was used to identify differentially expressed proteins in primary HUVECs among the three groups, after which Bioinformatics analysis was performed. Glucose uptake, ATP level, apoptosis, and differentially expressed proteins were assessed to investigate changes in energy metabolism.

**Results:**

A total of 8174 quantifiable proteins were detected, and 142 differentially expressed proteins were identified after comparing patients with GDM-D/GDM-I and healthy controls. Of the 142, 64 proteins were upregulated while 77 were downregulated. Bioinformatics analysis revealed that the differentially expressed proteins were involved in multiple biological processes and signaling pathways related to cellular processes, biological regulation, and metabolic processes. According to the results from KEGG analysis, there were changes in the PI3K/AKT signaling pathway after comparing the three groups. In addition, there was a decrease in glucose uptake in the GDM-I (*P* < 0.01) group. In GDM-I, there was a significant decrease in the levels of glucose transporter 1 (GLUT1) and glucose transporter 3 (GLUT3). Moreover, glucose uptake was significantly decreased in GDM-I, although in GDM-D, there was only a decrease in the levels of GLUT1. ATP levels decreased in GDM-I (*P* < 0.05) and apoptosis occurred in both the GDM-D and GDM-I groups. Compared to the normal controls, the levels of phosphate AKT and phosphate AMPK over total AKT and AMPK were reduced in the GDM-I group.

**Conclusion:**

In summary, endothelial dysfunction occurred in pregnancies with GDM even though the plasma glucose levels were controlled, and this dysfunction might be related to the degree of glucose tolerance. The energy dysfunction might be related to the regulation of the AKT/AMPK/mTOR signaling pathway.

**Supplementary Information:**

The online version contains supplementary material available at 10.1186/s12902-023-01305-6.

## Introduction

Gestational diabetes mellitus (GDM) is a serious pregnancy complication characterized by spontaneous maternal hyperglycemia and hyperinsulinemia. According to the World Health Organization (WHO), the global prevalence of GDM is slightly above 18% [1, 2]. In addition, epidemiological research has identified a series of risk factors for GDM, including obesity, westernized diets and micronutrient deficiencies, maternal age, and a family history of Polycystic Ovarian Syndrome or diabetes. GDM increases the risk of short-term and long-term health issues in both the mother and the fetus. Short-term complications include pre-eclampsia, operative delivery, shoulder dystocia, laceration of the birth canal, and macrosomia. On the other hand, long-term complications include hyperglycemia, diabetes, structural hypothalamic changes, and the risk of cardiovascular disease (CVD) [3–5]. It is recommended that treatment should start immediately after the diagnosis of GDM, with lifestyle changes (dietary modification and physical activity) being the first-line intervention. However, when lifestyle interventions fail to maintain optimal glucose levels, insulin or oral antidiabetics (Metformin or Sulfonylureas) are needed [6, 7]. While most short-term complications can be resolved by normalizing the maternal blood glucose levels during pregnancy, long-term complications cannot [8, 9]. Moreover, children from GDM mothers were shown to have a significantly higher BMI at 4–5 years compared to their normal counterparts [10]. The risk of being overweight and having metabolic syndrome was also reported to be 2–4 times higher in GDM offspring and insulin sensitivity as well as secretion were decreased [11]. According to recent research, 21% of offspring (18–27 years of age) born of GDM mothers had pre-diabetes or diabetes, which is an 8-fold increased risk compared to the background population [12]. Furthermore, children from GDM mothers were reported to have a higher systolic blood pressure in adolescence [13, 14] and a significantly higher risk of CVD later in life [15, 16].

The placenta, which acts as the connection between the mother and the fetus, plays a key role in sustaining fetal growth. Therefore, changes in either maternal or fetal circulation may alter placental structure and function with potential consequences for fetal development. Existing evidence shows that the placenta of GDM patients has increased vascular resistance and slow blood flow, with possible blood flow arrest leading to intrauterine growth distress and restriction of fetal growth [17]. Additionally, the placenta of GDM mothers was shown to have a significant increase in chorionic edema, fibrin deposition, calcification, and vascular congestion, which were strongly associated with the birth weight and rate of stillbirths in infants [18]. It is now believed that fetal programming for diseases in adulthood is strongly associated with the maternal-to-fetal circulation providing nutrients and other substances [19].

The endothelium is a thin layer of cells that lines the interior surface of blood vessels, and preservation of endothelial-cell function plays a critical role in the maintenance of maternal-to-fetal circulation. Nutrients, including glucose, lipids and amino acids are transferred from the maternal side through trophoblast, endothelium and finally into fetal circulation; and transport through the endothelium is a restricted process for its surface smaller than trophoblast’s [20]. Therefore, endothelial dysfunction may lead to disturbances in nutrient exchange from maternal to fetal circulation [21]. It was also reported, based on primary human umbilical vein endothelial cells (HUVECs), that GDM mothers with lifestyle changes gave birth to offspring with a lower metabolic activity [22]. However, comparisons and mechanisms of endothelial dysfunction in GDM patients under different types of therapy are limited. Notably, mass spectrometry-based proteomics has been widely applied in basic research and clinical diagnosis of human disease, involving analysis, characterization, and classification of all proteins in a genome. In this study, we compared primary HUVECs derived from normal pregnant women, pregnant women diagnosed with GDM and had received a lifestyle intervention (GDM-D), and pregnant women with GDM who were treated with insulin (GDM-I). A proteomic approach was employed to assess whether a GDM mother who received a different form of treatment underwent metabolic changes in HUVECs and to identify the underlying molecular pathways. Further identification studies were conducted based on the proteomics results. Assessment of pathological changes in the endothelium under different therapies may help clarify the pathology of long-term fetal complications associated with GDM mothers.

## Materials and methods

### Sample collection

This study was approved by the Ethical Committee of the First affiliated hospital of Kunming medical university. All pregnant women were sensitized before sample collection and provided written informed consent.

The inclusion criteria for all participants were as follows: Informed consent to participate in this study, individuals aged between 18 and 35 years, women between 38 and 41 weeks of gestation, singleton pregnancy, those without a diagnosis of diabetes mellitus, hypertension, kidney disease, and cardiovascular disease before pregnancy, and women not on any medication. Patients with GDM were diagnosed according to a 75-g oral glucose tolerance test (OGTT) at 24–28 weeks of gestation (fasting ≥ 5.1 mmol/L, 1 h ≥ 10.0 mmol/L, 2 h ≥ 8.5 mmol/L). The GDM-D group was treated with a lifestyle intervention and the GDM-I group received insulin therapy when lifestyle management failed. All the management decisions were based on existing guidelines [6, 23].

HUVECs were obtained from normal pregnant women (n = 11), GDM-D (n = 14) and GDM-I (n = 12) patients. Umbilical cords (10–15 cm) were cut within 30 min after birth and stored immediately in saline at 4 ℃. The umbilical cords were then washed of the residual blood after which endothelial cells were separated from the lumens of human umbilical veins with a 1% collagen enzyme solute (Worthington, United States) for 30 min at 37 ℃. After digestion, the cells were cultured in endothelial cell medium (ECM) (ScienCell, United States) and incubated at 37 ℃ in 5% CO2 using standard cell culture techniques. After reaching a confluence of 80%, the cells were detached using 0.25% trypsin– EDTA. The cells used in this experiment were subjected to three to four passages [24].

### Protein extraction

HUVECs collected from normal pregnant women (n = 3), GDM-D (n = 3) and GDM-I (n = 3) patients, and cultured to passage 3–4 respectively, were sonicated three times on ice using a high intensity ultrasonic processor (Scientz) in lysis buffer (8 M urea, 1% Protease Inhibitor Cocktail). The remaining debris was removed by centrifugation at 12,000 g and 4 °C for 10 min. The supernatants were quantified for their protein concentrations using the BCA protein Assay Kit (Beyotime Institute of Biotechnology, China).

### Protein digestion and TMT labelling

The digestion of proteins was based on the published method [25, 26]. The supernatants were reduced with 5 mM dithiothreitol for 30 min at 56 °C and alkylated with 11 mM iodoacetamide for 15 min at room temperature, in the dark. The protein samples were then diluted by adding 100 mM TEAB to a urea concentration of less than 2 M. Finally, trypsin was added at a 1:50 trypsin-to-protein mass ratio for the first digestion overnight and a 1:100 trypsin-to-protein mass ratio for a second 4 h-digestion. Finally, the peptides were desalted using the C18 SPE column. Tryptic peptides were firstly dissolved in 0.5 M TEAB. Each channel of peptide was labeled with their respective TMT reagent (ThermoFisher Scientific), and incubated for 2 h at room temperature. Five microliters of each sample were pooled, desalted, and analyzed through MS to check the labeling efficiency. After the labeling-efficiency check, samples were diluted by adding 5% hydroxylamine. The pooled samples were then desalted with the Strata X C18 SPE column (Phenomenex) and dried through vacuum centrifugation.

Specifically, three HUVECs samples from normal pregnant women were labelled with the 126 N isobaric TMT tag, while samples from patients with GDM were labelled with the 127 N tag. The other three syncytiotrophoblast samples from PE patients with GDM were labelled with the 129 C, 130 N, and 130 C isobaric TMT tags. After TMT labelling, the digested sample was separated into 10 fractions, and excess label and salts were removed according to the Pierce High pH Reversed-Phase Fractionation Kit Manual (Thermo Fisher Scientific).

### UPLC-MS/MS analysis

The UPLC-MS/MS analysis was based on the published method [27]. The method of UPLC-MS/MS was referred to the protocol supplied by PTMbio Co. (Hangzhou, China). The tryptic peptides were dissolved in 0.1% formic acid (solvent A) then directly loaded onto a home-made reversed-phase analytical column (15-cm length, 75 μm i.d.). The gradient consisted of an increase in solvent B (0.1% formic acid in 98% acetonitrile) from 6 to 23% over 26 min, 23–35% in 8 min, an increase to 80% in 3 min then holding at 80% for the last 3 min, all at a constant flow rate of 400 nL/min on an EASY-nLC 1000 UPLC system. The peptides were subjected to an NSI source followed by tandem mass spectrometry (MS/MS) in the Q Exactive^™^ Plus (Thermo) coupled online to the UPLC. The electrospray voltage applied was 2.0 kV while the m/z scan range was 350 to 1800 for a full scan. Intact peptides were detected in the Orbitrap at a resolution of 70,000. Peptides were then selected for MS/MS using an NCE setting of 28 and the fragments were detected in the Orbitrap at a resolution of 17,500. A data dependent procedure that alternated between one MS scan followed by 20 MS/MS scans with a 15.0s dynamic exclusion was used. The automatic gain control (AGC) was set at 5E4 while the fixed first mass was set as 100 m/z.

### Protein identification and bioinformatic analysis

To identify differentially expressed proteins, the fold change was set at > 1.2 or < 0.83 with a *P* value < 0.05 (Student’s t-test). The gene ontology (GO) annotation of differentially expressed proteins was blasted against the SwissProt database (human) using the NCBI BLAST + client software. Functional annotation was searched against the online Kyoto Encyclopedia of Genes and Genomes (KEGG) database (http://geneontology.org/) [28–30]. GO enrichment and KEGG pathway enrichment analyses were conducted based on the Fisher’ exact test. Hierarchical clustering was performed using Cluster 3.0 (http://bonsai.hgc.jp/~mdehoon/software/cluster/software.htm) and the Java Treeview software.

### Immunohistochemistry

Umbilical cord tissues from the three groups were fixed in 10% formalin, followed by tissue processing and embedding in paraffin wax. Four-micron thick sections were made on polylysine coated slides for the immunohistochemical procedures. Firstly, sections were dewaxed then rehydrated and antigen retrieval was performed in a microwave oven for 15 min. Thereafter, the slides were treated with a 3% hydrogen peroxide block to remove endogenous peroxidase activity. After washing with PBS, enough drops of primary antibody for GLUT1 (Servicebio, rabbit polyclonal antibody), GLUT3 (Cellsignal, rabbit monoclonal antibody), p-AMPKα (Abcam, rabbit monoclonal antibody), p-AKT (Cellsignal, rabbit monoclonal antibody), and cleaved caspase-3 (Cellsignal, rabbit monoclonaln antibody) were separately added and incubated for 1 h. The secondary antibody was added thereafter. Reaction was visualized with DAB and the slides were counterstained with hematoxylin. Additionally, the acquisition of tissue staining was visualized under a microscope. The intensity of slides was finally assessed and calculated using H-scores through AI analysis from Servicebio Ltd.

### Glucose uptake and ATP assay

A total of 5 × 10^3^ HUVECs collected from the three groups (n = 6 per group) were cultured separately in 96-well plates, for 48 h. The assay was conducted using the Glucose uptake-GloTM Kit (Promega, USA), following the manufacturer’s instructions. ATP assessment was performed in a similar manner to the procedure above and the assay was conducted using the CellTiter-Glo Luminesent Cell Viability Assay (Promega, USA) following the manufacturer’s instructions.

### Apoptosis analysis

Flow cytometry analysis for cell apoptosis in the HUVECs was conducted using the Coralite 488-Annexin V and PI apoptosis detection kit (Proteintech, USA) and the method was based on the published paper [31]. Cells from each group were collected, rinsed with cold PBS, and stained using Coralite 488-Annexin V/PI. The cells were then immediately detected using an Accuri C6 flow cytometer (BD Biosciences, United States). Data were analyzed using the FlowJo software (ver.10.0, FlowJo LLC, United States).

### Western blotting

Cells were lysed in lysis buffer (Beyotime Institute of Biotechnology, China) supplemented with 1 mM PMSF with or without 0.01 mM phosphatase inhibitors. Protein concentration was determined using the BCA protein assay (Beyotime Institute of Biotechnology, China). Five micrograms of protein from each sample were separated using 10–12% SDS-PAGE then electro-transferred onto PVDF membranes (Millipore, United States) for immunoblotting analysis. The PVDF membranes were cut depending on the molecule and the original blots are presented as supplementary data. The primary antibodies included: anti-GAPDH (1:500, ab108319, Abcam), anti-GLUT1 (1:5000, ab115730, Abcam), anti-GLUT3 (1:2000, 40,538, Cellsignal), and anti-pAMPKα (Thr172, 1:1000, ab6276, Abcam), anti-AMPK (1:1000, ab6276, Abcam), anti-p4EBP1 (1:2000, 2855T, Cellsignal), anti-4EBP1(1:1000, 9644 S, Cellsignal), anti-pmTOR(1:1000, 5536T, Cellsignal), anti-mTOR(Ser2448) (1:1000, 2983T, Cellsignal), anti-Cleaved PARP (1:1000, 5625 S, Cellsignal), anti-cleaved caspase-3 (1:1000, 9554 S, Cellsignal), anti-phosphate AKT(Ser473) (D9E) (1:500, 4060, Cellsignal), and anti-AKT (1:1000, R23412, Zenbio). Protein bands were visualized using the enhanced chemiluminescence system (Pierce, Rockford, IL) then analyzed in the ImageJ software. The protein levels were normalized to the levels of GAPDH and the fold change relative to the normal group was calculated in every experiment.

### Data analysis

Data were analyzed using the Graphpad (v.13.0.0; SPSS Inc., United States) statistical software and are presented as the means ± standard deviation. All experiments were repeated at least three times. Two-tailed Student’s t-tests and one-way ANOVA were used to compare the results, which were considered statistically significant at *P* < 0.05.

## Results

The characteristics of the study populations are summarized in Table 1. The comparisons were made with one-way ANOVA. No differences were observed in the maternal ages, BMI, and systolic/diastolic blood pressure among the groups. However, the GDM-I category had higher blood glucose values both at fasting and 1-hour postprandial, compared to the normal and GDM-D groups. After lifestyle intervention or insulin therapy, the fasting plasma glucose levels were managed at the target values with no significant differences.

**Table 1:** Maternal and perinatal characteristics of women with normal, diet-treated (GDM-D) or insulin-treated (GDM-I) gestational diabetes

	Normal (n = 11)	GDM-D (n = 14)	GDM-I (n = 12)	*P* value
Maternal age (years)	30.3 ± 2.9	31.6 ± 4.3	31.6 ± 3.6	0.639
Maternal BMI (kg/m2)	25.64 ± 2.29	26.32 ± 4.53	29.25 ± 5.55	0.131
Systolic blood pressure (mmHg)	116.1 ± 8.6	118.5 ± 9.1	123.6 ± 16.3	0.324
Diastolic blood pressure (mmHg)	76.8 ± 10.3	75.5 ± 10.4	82.3 ± 10.6	0.245
75g OGTT				
Fasting plasma glucose (mmol/L)	4.44 ± 0.26	5.06 ± 0.56	5.28 ± 1.53**##	0.0007
1-hour plasma glucose (mmol/L)	8.17 ± 1.42	8.34 ± 2.04	9.75 ± 2.06	0.138
2-hour plasma glucose (mmol/L)	6.30 ± 0.78	7.17 ± 0.85	8.36 ± 1.41**#	0.0008
Fasting plasma glucose after intervention (mmol/L)		4.55 ± 0.65	4.92 ± 0.47	0.573

one-way ANOVA was used for statistics among the three groups; ∗*P* < 0.05, ∗∗*P* < 0.01 compared with normal; #*P* < 0.05, ##*P* < 0.01 compared with GDM-D;

### Energy metabolism was abnormal in HUVECs from both GDM-D and GDM-I

**Fig. 1:**
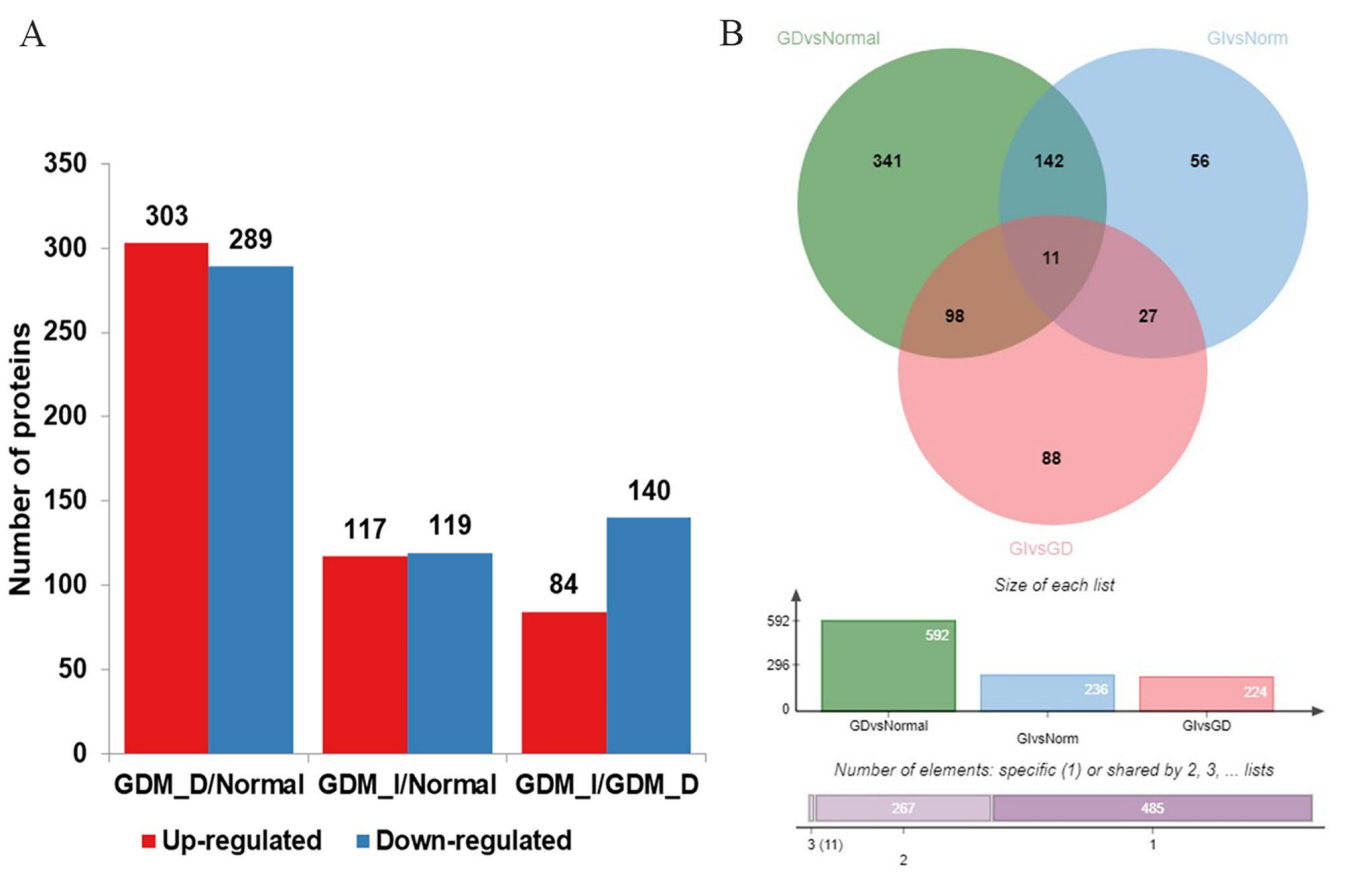
The differentially expressed proteins were found in every two groups

**Fig. 2:**
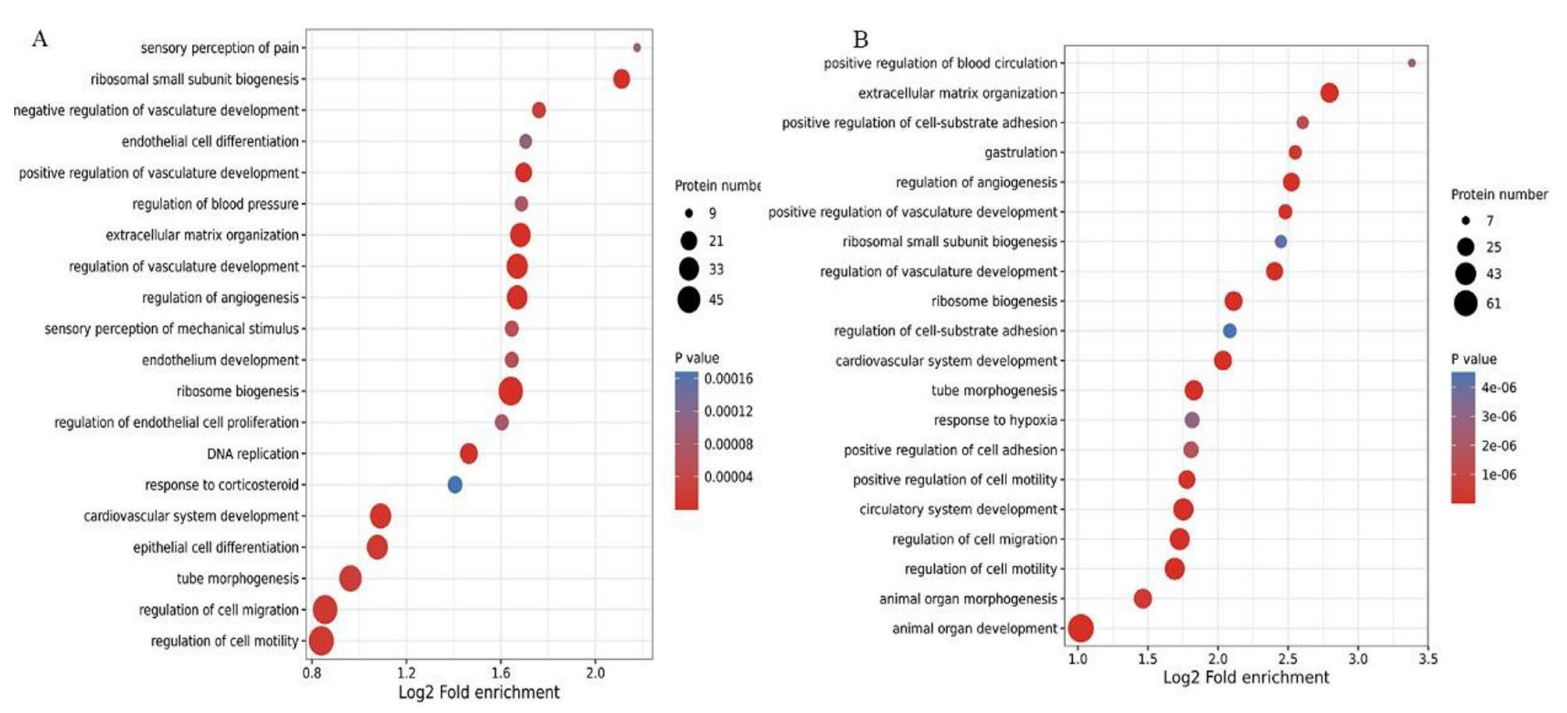
Bubble diagram of enrichment and distribution of DEPs in Biological process Note: (A) Comparison between GDM-D and Normal. (B) Comparison between GDM-I and Normal

**Fig. 3:**
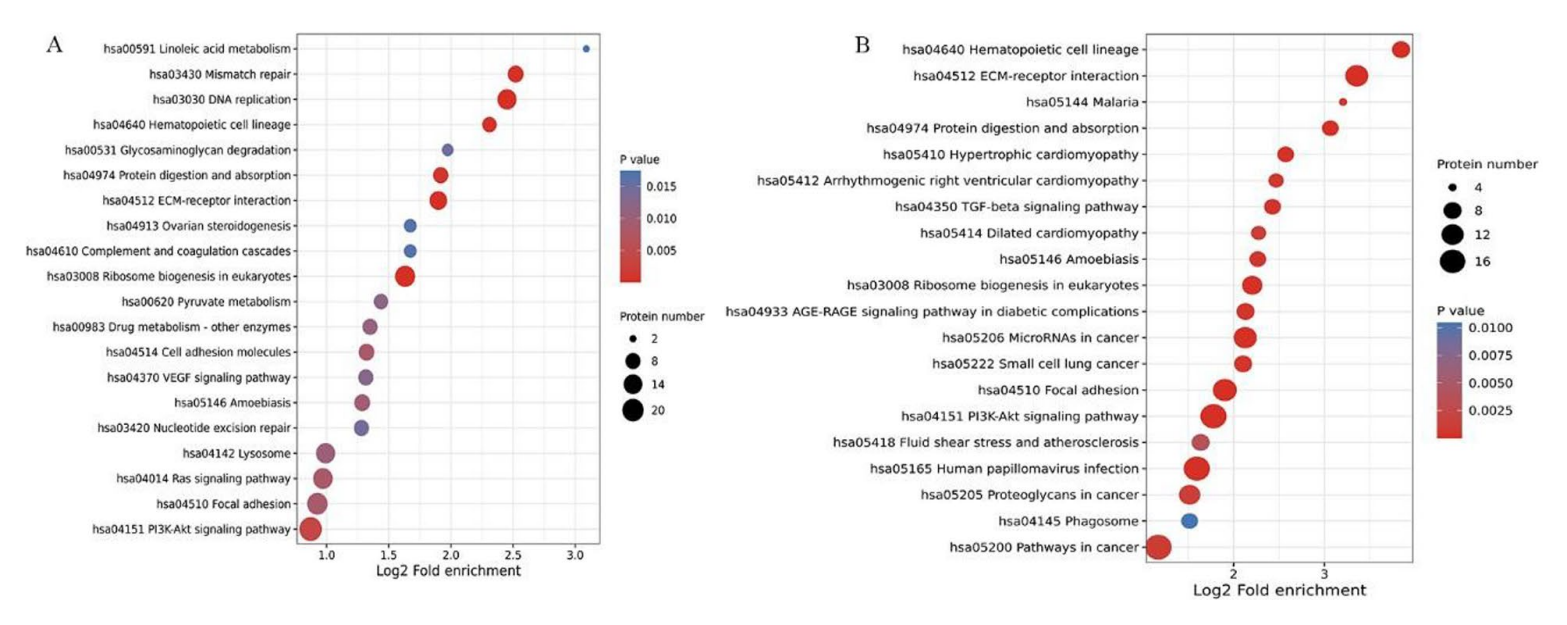
Bubble diagram illustrating the enrichment and distribution of DEPs in the KEGG pathway analysis Note: (A) Comparison between GDM-D and Normal. (B) Comparison between GDM-I and Normal

To discover plasma protein alternations induced by different interventions, global proteomic profiling of HUVECs from normal, GDM-D, and GDM-I participants was conducted through LC-MS/MS using TMT labeling. High-accuracy LC-MS/MS was used to identify and quantitatively detect a large scale of proteins. A total of 176,297 unique peptides and 8174 proteins were identified in all the samples. The differentially expressed proteins were then identified in every two groups (Fig. 1-A). According to the protein identification criteria, 142 differentially expressed proteins were found between GDM-D/GDM-I patients and healthy controls. Among the 142 proteins, 64 were upregulated while 77 were downregulated (Fig. 1-B). In terms of biological process, compared to the normal controls, DEPs in both the GDM-D and GDM-I groups were mainly enriched in organ development, cellular mitosis, cell migration, cell adhesion, and response to hypoxia (Fig. 2). Compared to the normal controls, DEPs from the GDM-D group were mainly enriched in such pathways as PI3K/AKT signaling, focal adhesion, Ras signaling, Lysosome, and VEGF signaling (Fig. 3-A). On the other hand, DEPs in the GDM-I group were mainly enriched in the pathways of cancer, Human papillomavirus infection, PI3K/AKT signaling, focal adhesion and the AGE − RAGE signaling pathway in diabetic complications. (Fig. 3-B). These results from DEPs analysis showed significant changes in energy metabolism under pathology and different therapies.

### Glucose uptake and ATP level were lower in GDM-D/GMD-I pregnancies than in normal controls

**Fig. 4:**
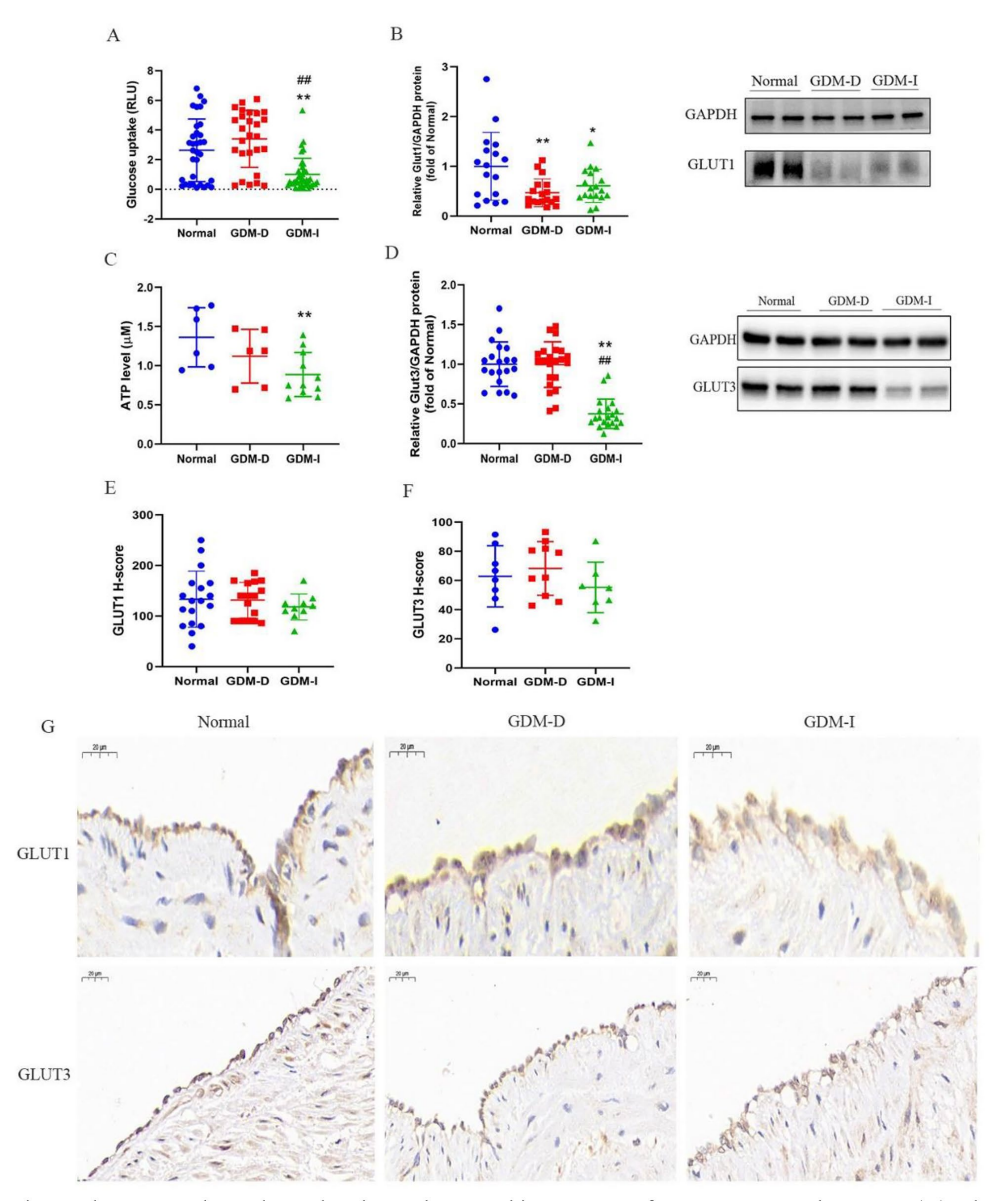
Glucose uptake and ATP level was decreased in HUVECs from GDM-D and GDM-I. (A) Glucose uptake was determined in HUVECs from Normal (n = 6), GDM-D (n = 7), and GDM-I (n = 7). Western blot analysis showing expression level of GLUT1 (n = 9 per group) (B) and GLUT3 (n = 10 per group) (D). ATP levels in HUVECs from Normal (n = 6), GDM-D (n = 6), GDM-I (n = 11). H-scores of GLUT1 and GLUT3 in immunohistochemistry test (E, F). Representative photographs of immunohistochemistry staining in the indicated groups (G). Data are expressed as the means ± SD. The results were analyzed with one-way ANOVA test. (∗*P* < 0.05, ∗∗*P* < 0.01 compared with normal; #*P* < 0.05, ##*P* < 0.01 compared with GDM-D)

**Fig. 5:**
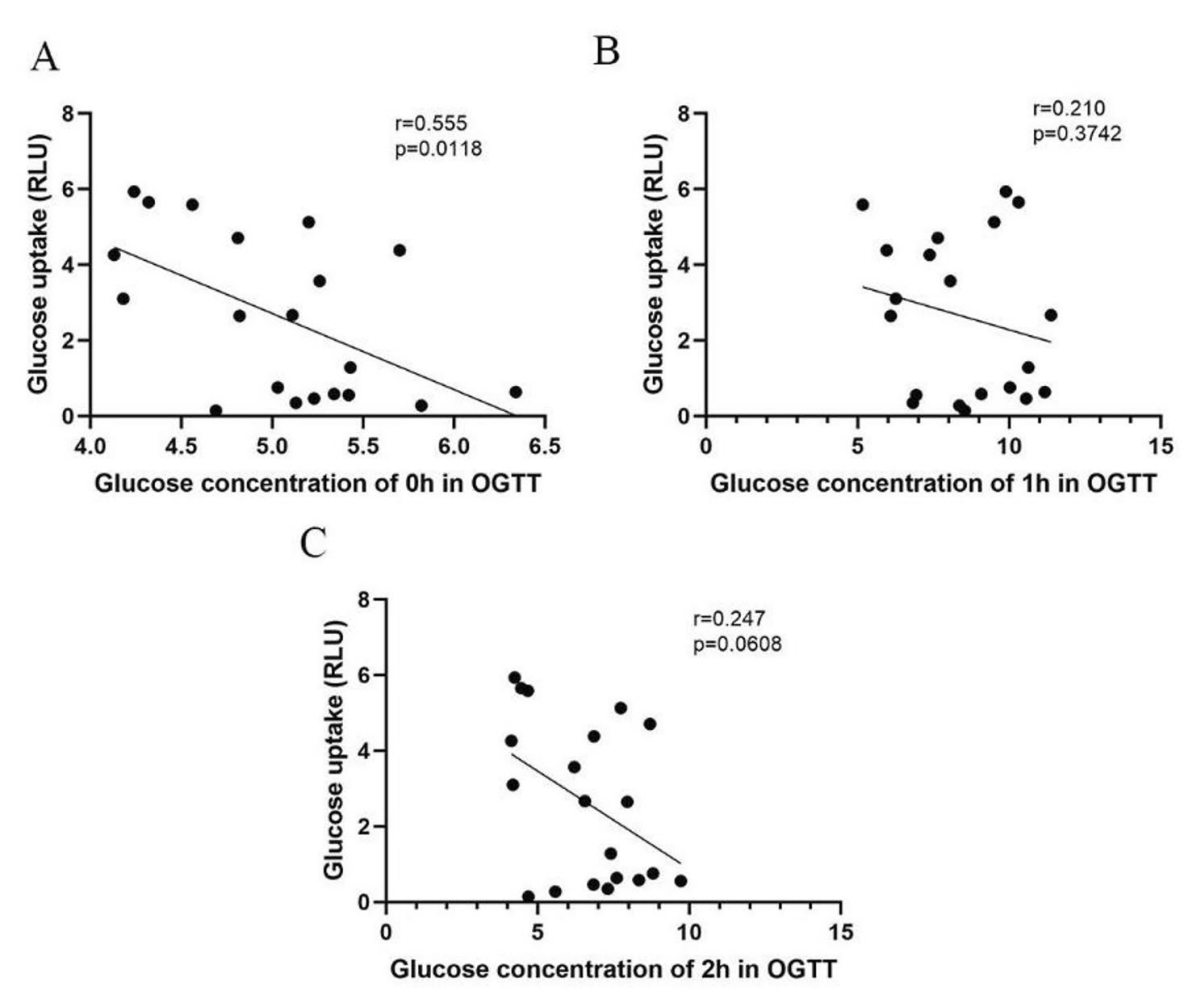
Correlation analysis between glucose uptake of HUVECs and OGTT fasting glucose (A), 1-hour postprandial glucose (B), and OGTT 2-hour postprandial glucose (C)

Glucose is the main energy source for fetal growth and development, and since the fetus is unable to synthesize glucose, it depends on transplacental glucose from the mother [32]. It is unclear whether GDM can affect the amount of glucose transported into fetoplacental circulation. Results from proteomic analysis showed that the expression of GLUT1 was significantly lower in both the GDM-D or GDM-I groups. GLUT1 is a membrane transporter that belongs to the glucose transporter proteins (GLUTs) family (with 12 isoforms), whose members are responsible for the transmembrane transport of glucose into tissues. GLUT1 is abundantly present in humans while GLUT3 has the highest affinity for glucose [33, 34]. To validate the proteomics results, we used Western blotting and immunohistochemical techniques to analyze the expression levels of GLUT1 and GLUT3 in both HUVECs and umbilical cord tissues from normal pregnant women and GDM patients. The findings revealed that compared to the normal controls, the levels of GLUT1 were significantly lower in the GDM group (including GDM-D and GDM-I). In addition, the levels of GLUT3 were significantly lower in GDM-I compared to the normal group (*P* < 0.01), but the GDM-D category showed no significant difference (Fig. 4). To validate this finding, we performed immunohistochemistry analysis of GLUT1 and GLUT3 in umbilical cord tissues. The H-scores were calculated and there were no differences in GLUT1 (133.3 ± 55.51 (Normal) vs. 131.6 ± 35.11 (GDM-D) vs. 118.0 ± 25.52 (GDM-I) and GLUT3 (62.86 ± 21.02 (Normal) vs. 68.21 ± 18.41 (GDM-D) vs. 55.27 ± 17.33 (GDM-I)) among the three groups (Fig. 4-E and -F). The typical diagrams are shown in Fig. 4-G. In the glucose uptake and ATP tests, we observed that, compared to the normal controls, HUVECs from GDM-I patients had lower glucose uptake rates than those from both the normal and GDM-D groups (Fig. 4-A). Moreover, the ATP levels in HUVECs from GDM-D and GDM-I patients were significantly decreased, relative to the normal controls (*P* < 0.05 and *P* < 0.01 respectively, Fig. 4-B). Additionally, the relationships between glucose uptake and blood glucose levels (before clinical intervention) were analyzed, and the results showed that the degree of glucose uptake was significantly related to the fasting glucose levels (*P* = 0.0118), although there were no differences between the degree of glucose uptake and 1-hour or 2-hour glucose levels (Fig. 5).

### Cell apoptosis was increased in HUVECs from GDM-D and GDM-I

**Fig. 6:**
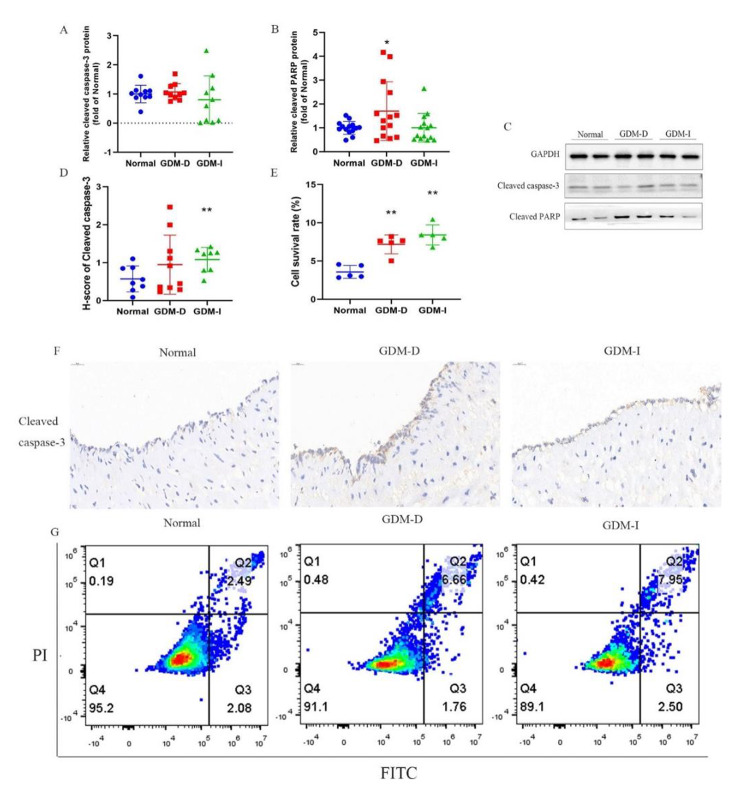
The apoptosis of HUVECs from GDM-D and GDM-I. Western blot analysis of cleaved caspase-3 (A, n = 5 per group), cleaved PARP (B, n = 9 per group) expression and representative membrane blots (C). H-scores of cleaved caspase-3 and representative photographs of immunohistochemistry staining in the indicated groups (E and F). Annexin V/PI assay of HUVECs from Normal (n = 3), GDM-D (n = 3) and GDM-I (n = 3) (E and G). Histograms showing the apoptosis rates in the indicated groups. The results were analyzed with one-way ANOVA test. (∗*P* < 0.05, ∗∗*P* < 0.01 compared with normal; #*P* < 0.05, ##*P* < 0.01 compared with GDM-D).

There is evidence that hyperglycemic can cause endothelial cell apoptosis [35]. In this study, the results showed that the levels of ATP from the GDM-I group were significantly lower than those in the normal controls, indicating possible mitochondrial dysfunction. According to existing evidence, mitochondrial dysfunction is an early cause of endothelial injury [36]. Flow cytometry was additionally used to assess cell apoptosis and the apoptosis-inducing factors, cleaved caspase-3 and cleaved PARP were analyzed. The findings revealed that HUVECs from GDM-D and GDM-I patients had undergone significant apoptosis (Fig. 6-E). The representative plots are shown in Fig. 6-G. However, there was no difference in the levels of cleaved caspase-3 among the three groups (Fig. 6-A). Western blot analysis revealed that compared to the normal controls, the expression of cleaved PARP was higher in the GDM-D group (*P* < 0.05, Fig. 6-B). Representative blots are shown in Fig. 6-C. Moreover, immunohistochemical analysis of cleaved caspase-3 was conducted, and the H-scores from the GDM-I category were significantly higher than those from the normal group (0.57 ± 0.34 (Normal) vs. 0.95 ± 0.78 (GDM-D) vs. 1.08 ± 0.32 (GDM-I), Fig. 6-D). The typical diagrams are shown in Fig. 6-F.

### AKT/AMPK-mTOR pathway was changed in HUVECs from GDM-D and GDM-I

**Fig. 7:**
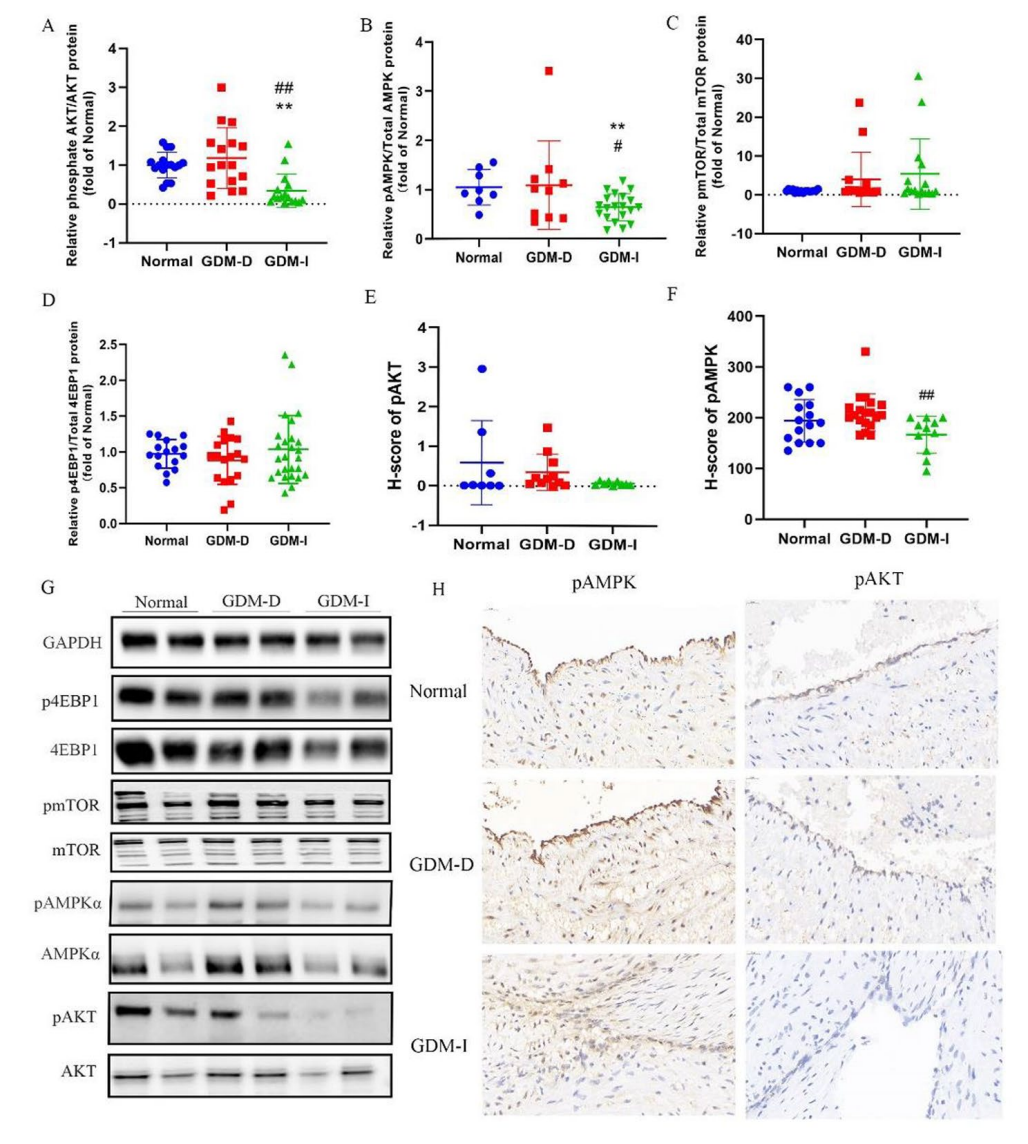
AKT/AMPK/mTOR pathway was altered in HUVECs from GDM-D and GDM-I. Western analysis showing expression levels of pAKT, AKT, pAMPK, AMPKα, p4EBP1, 4EBP1, pmTOR, mTOR and Histograms displaying the relative pAKT/AKT (A, n = 8 per group), pAMPKα/AMPKα (B, with normal n = 4, GDM-D n = 5, GDM-I, n = 10), pmTOR/mTOR (C, n = 9 per group), p4EBP1/4EBP1(D, n = 9 per group). H-scores of pAKT and pAMPK in immunohistochemistry test (E, F). Representative photographs of immunohistochemistry staining of pAMPKα and pAKT in the indicated groups (H). (∗*P* < 0.05, ∗∗*P* < 0.01 compared with normal; #*P* < 0.05, ##*P* < 0.01 compared with GDM-D).

According to the proteomics results, the three groups differed in energy metabolism and cell growth. On the other hand, KEGG analysis showed that the three groups differed in the PI3K/AKT pathway. AKT is a molecule that is involved in many cellular processes including cell survival, cell cycle progression and metabolism [37]. PI3K is found upstream of AKT and ligands such as insulin or growth factors can activate PI3K through G-protein coupled receptors. Activated PI3K phosphorylates the substrate phosphatidylinositol 4,5-biphosphate (PIP2) to generate phosphatidylinositol 3,4,5-triphosphate (PIP3), subsequently recruiting signaling proteins, including AKT [38]. AKT further phosphorylates the downstream mTOR by reducing the inhibitory effects of the tuberous sclerosis complex (TSC1/TSC2) on mTORC1 [39]. Notably, mTORC1 can regulate several effectors proteins through phosphorylation, including the eukaryotic translation initiation factor-4E (eIF4E)-binding protein-1(4EBP1) to modulate apoptosis or increase glycogen synthesis [40]. AMPK is an important molecule that senses changes in growth factors, nutrient signals, and metabolic stress in cells, containing a catalytic subunit (α) and two regulatory subunits (β and γ). During inhibition of ATP production, AMPK is activated and it directly phosphorylates TSC2 or the critical mTORC1 binding subunit, Raptor, thus inhibiting mTORC1 activity [40]. Here, we observed that the levels of ATP were lower in the GDM-I group but the change of signaling pathway was unknown. We measured the levels of phosphorylated and total forms of AKT, AMPKα, mTOR, 4EBP1 in HUVECs obtained from normal, GDM-D and GDM-I pregnant women. The ratios of phosphorylated to total forms of AKT, AMPKα, mTOR and 4eBP1 were calculated and analyzed through one-way ANNOVA. The results showed that HUVECs from the GDM-I category had a significantly lower phosphorylated to total AKT and AMPKα ratio compared to those from the normal and GDM-D groups (Fig. 7-A, B). There was however no difference in phosphorylated to total mTOR and 4eBP1 among the three groups (Fig. 7-C and -D). Representative blots are shown in Fig. 7-G. Furthermore, we assessed the levels of pAMPKα and pAKT in umbilical cord tissues through immunochemical tests. According to the calculated H-scores, the levels of pAMPKα in umbilical cord tissues from GDM-I patients were significantly lower (194.0 ± 41.8 (Normal) vs. 210.5 ± 36.6 (GDM-D) vs. 166.8 ± 36.4 (GDM-I), Fig. 7-E). In contrast, there was no difference in the levels of pAKT in umbilical cord tissues from the three categories (0.588 ± 1.07 (Normal) vs. 0.350 ± 0.459 (GDM-D) vs. 0.07 ± 0.05 (GDM-I), Fig. 7-F). Representative immunohistochemistry pictures are shown in Fig. 7-H.

## Discussion

The global prevalence of GDM has been on the rise over the recent years [4]. Despite lifestyle changes or insulin treatment, long-term complications in offspring from GDM mothers are still prevalent. Fetal development and growth are jointly facilitated by multiple physiological factors. In addition, the placental endothelium plays a crucial role in ensuring proper circulation and nutrient transport in the fetoplacental system. It is known that hyperglycemia triggers multiple forms of endothelial dysfunction. However, there is limited knowledge on endothelial function in the placenta during a GDM pregnancy. In this study, GDM pregnant women were divided into two groups based on the clinical intervention i.e., lifestyle changes or insulin treatment. According to the OGTT results, pregnant women from the GDM-I group had significantly higher levels of fasting and 2-hour blood glucose, suggesting that GDM-I might result in a more serious dysfunction in glucose tolerance.

We compared the proteomics of HUVECs obtained from normal, GDM-D, and GDM-I pregnant women and identified GLUT1 as one of the DEPs while the PI3K/AKT pathway was significantly different among the three groups. Since the fetus cannot synthesize glucose, the glucose transferred by GLUTs from the mother to the fetus in the placenta is critical for fetal development [41]. Notably, GLUT1 is abundant in the placenta while GLUT3 is considered to be the highest affinity glucose transporter [42]. Both of these (GLUT1 and GLUT3) were expressed in the trophoblast and endothelium of the placenta. Although the impact of GDM on glucose uptake and various GLUTs has been investigated in the trophoblast or tissues from GDM placenta [43, 44], the levels of GLUT1 and GLUT3 expressed in the placental endothelium from GDM under different treatments, are largely unknown. We for the first time provide evidence that the levels of GLUT1, GLUT3 and glucose uptake were decreased in the endothelia of GDM women receiving insulin therapy. Although there was a decrease in the level of GLUT1 in GDM-D, there is no difference in glucose uptake between normal and GDM-D cells, indicating that GLUT3 might partially compensate for the effect of GLUT1 in the GDM-D group. It is reported that the surface area of the maternal trophoblast side is nearly 5-fold bigger than that of the fetal endothelial side [45]. Therefore, we speculated that compared with GLUT1, GLUT3 expressed on the endothelium was more important in transferring glucose through the placenta. Regarding results from the relationship between the degree of glucose uptake and OGTT, despite controlling the blood glucose levels, regulation of GLUTs was still related to dysfunction in maternal glucose tolerance before clinical treatment.

There is evidence that high glucose levels enhance cell apoptosis by suppressing AMPK and AKT in endothelial cells [35, 46]. Here, we found that apoptosis was enhanced in HUVECs from both GDM-D and GDM-I placentas, while ATP levels were reduced in GDM-I. Mitochondria are the primary sources of cellular energy, involved in not only metabolic processes and respiration but are also responsible for ATP production, control of cell metabolism and apoptosis regulation. It is reported that GDM affects the mother and the human fetoplacental unit, resulting in mitochondrial dysfunction [47, 48]. The decreased ATP levels suggest that mitochondrial dysfunction might have occurred in the placental endothelia from GDM patients, which could trigger apoptosis by activating cytochrome C, caspase-3 and PARP [36]. According to our data, there was a significant increase in the levels of cleaved caspase-3 and PARP in GDM-I and GDM-D, respectively, which might be related to the complex mechanism of apoptosis. Apoptosis is mediated by several signaling pathways. In addition to the mitochondrial pathway, endoplasmic reticulum (ER) stress also contributes to apoptosis. Notably, ER stress is increasingly getting recognition as an important mechanism in the development of diabetes [49] and is associated with endothelial dysfunction in resistance vessels. Nonetheless, it is unclear whether these mechanisms also contribute to diabetes-mediated endothelial dysfunction. It is possible that the apoptosis of HUEVCs from GDM patients might be linked to the mitochondrial pathway.

AMPK and AKT are two primary effectors during response to metabolic stress, and both can regulate glucose uptake and cell apoptosis. Although it is well known that AMPK/AKT plays a role in the pathophysiology of GDM [50, 51], the reported changes in AMPK/AKT in the placenta of GDM patients are inconsistent. For instance, some studies reported that the level of pAKT was decreased in placental tissues from GDM women while others documented increased levels of phosphate AKT [52, 53]. The reported changes in phosphate AMPK levels in the placentas of GDM women are also conflicting. Guo Yao et al. showed that women with GDM had lower placental levels of AMPK and pAMPK [54], contrary to other reports [55]. These inconsistent results might be because these previous studies mainly focused on placental tissues consisting of trophoblasts and endothelial cells, both of which might have different pathologies in GDM development. Furthermore, these studies did not differentiate the treatment or the degree of severity in glucose tolerance in the GDM participants they included. In our study, we for the first time, present evidence of the changes in AKT/AMPK in primary cells obtained from GDM patients treated with either a lifestyle intervention or insulin. Consistent with proteomic and glucose uptake results, the levels of phosphate AKT over total AKT and total AMPK were also downregulated in HUVECs from both GDM-D and GDM-I. Decreased AMPK/AKT can modulate glucose uptake for the transcription of GLUTs through the transcription factor, CREB [42]. The translocation of GLUTs from the cytosol to the membrane was also affected by the activity of the AMPK signaling pathway [44]. Here, there were no differences of the levels of phosphorylated over total forms of mTOR, 4EBP1 of HUVECs from GDM-I group. Although mTOR is modulated by both AMPK and AKT, AKT and AMPK likely have independent effects on mTOR phosphorylation under hyperglycemic conditions [56]. Furthermore, the final levels of pmTOR were dependent on the intracellular environment.

In conclusion, this study shows differential changes in glucose uptake/metabolism, apoptosis and the levels of total and phosphate AKT, AMPK, mTOR, 4eBP1 using primary HUVECs obtained from GDM women under different treatments. We found that AKT/AMPK and glucose uptake were downregulated while apoptosis was enhanced in GDM patients receiving insulin therapy. In addition, placental endothelial dysfunction adversely affected placental circulation and nutrient exchange. Despite controlling the blood glucose levels, the dysfunction still occurred. These pathological changes might be related to the long-term complications arising from GDM in pregnancy, and further mechanistic studies are needed.

## Electronic supplementary material

Below is the link to the electronic supplementary material.

Supplementary Material 1

## Data Availability

The raw data generated and analyzed in this study are not publicly available due to the appropriate protection of patients’ personal information, but are available from the corresponding author on a reasonable request.
