# Supplementary material for: Feto-placental endothelial dysfunction in Gestational Diabetes Mellitus under dietary or insulin therapy
Source: BMC Endocr Disord. 2023 Feb 23;23:48. doi: 10.1186/s12902-023-01305-6 (PMC9948408; doi:10.1186/s12902-023-01305-6)
Supplement: Supplementary file 1 — Supplementary Material 1 [file 12902_2023_1305_MOESM1_ESM.pdf]

In the page bellowed, the original blots of GLUT1 and GAPDH were shown. We analyzed normal, GDM-D, and GDM-I (n=9 per group with duplicate tests), respectively. The levels of GLUT1 (left side) were normalized to the levels of GAPDH (right side) and the fold change relative to the normal group was calculated in every experiment. Finally, the data were statistics in Fig. 4-B and the blot used in the manuscript is denoted with red lines.

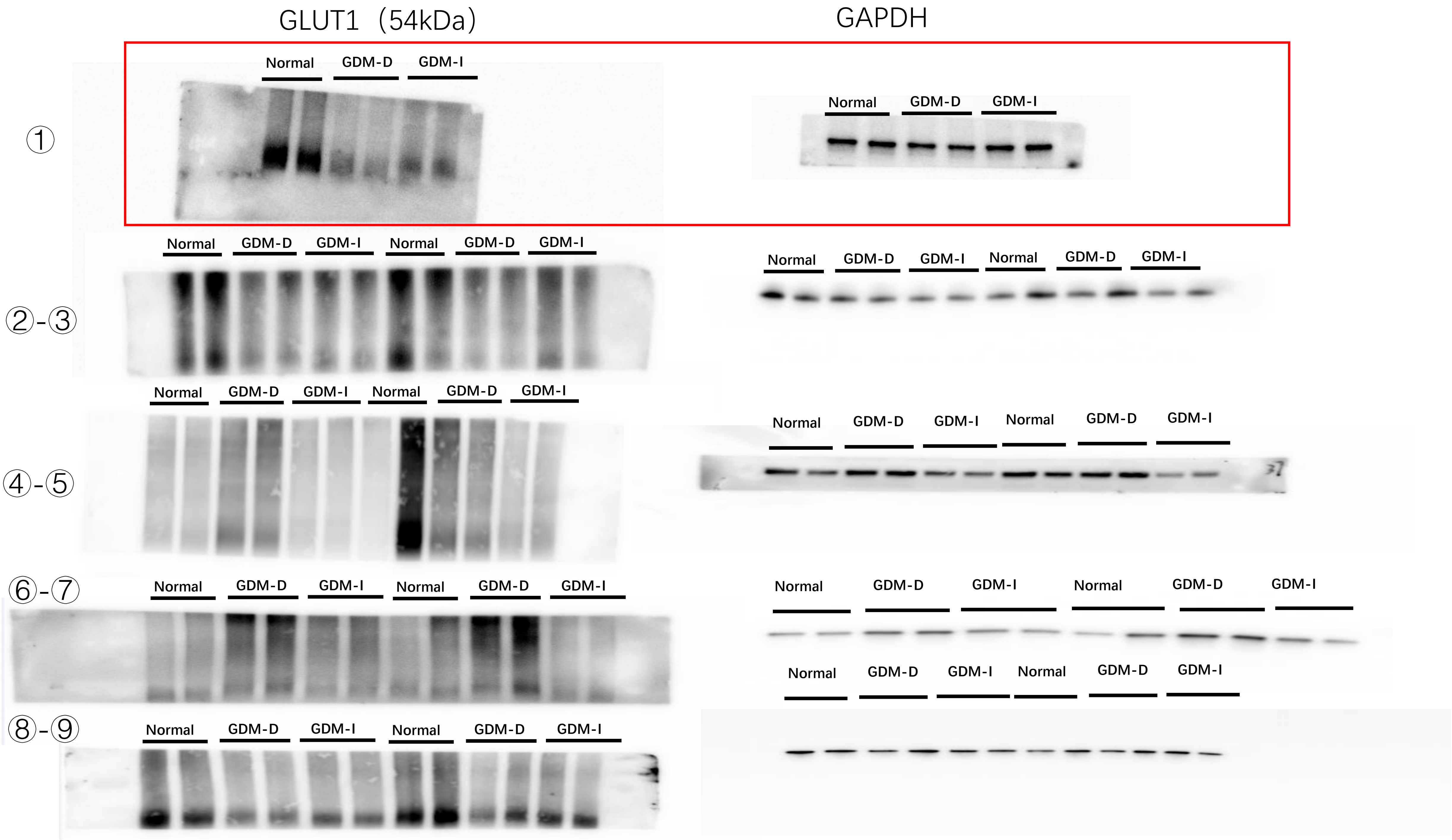

GLUT1（54kDa）
GAPDH
①
Normal GDM-D GDM-I
Normal GDM-D GDM-I
②-③
Normal GDM-D GDM-I Normal GDM-D GDM-I
Normal GDM-D GDM-I Normal GDM-D GDM-I
④-⑤
Normal GDM-D GDM-I Normal GDM-D GDM-I
Normal GDM-D GDM-I Normal GDM-D GDM-I
⑥-⑦
Normal GDM-D GDM-I Normal GDM-D GDM-I
Normal GDM-D GDM-I Normal GDM-D GDM-I
⑧-⑨
Normal GDM-D GDM-I Normal GDM-D GDM-I
Normal GDM-D GDM-I Normal GDM-D GDM-I

In the page bellowed, the original blots of GLUT3 and GAPDH were shown. We analyzed normal, GDM-D, and GDM-I (n=10 per group with duplicate tests), respectively. The levels of GLUT3 (left side) were normalized to the levels of GAPDH (right side) and the fold change relative to the normal group was calculated in every experiment. Finally, the data were statistics in Fig. 4-D and the blot used in the manuscript is denoted with red lines.

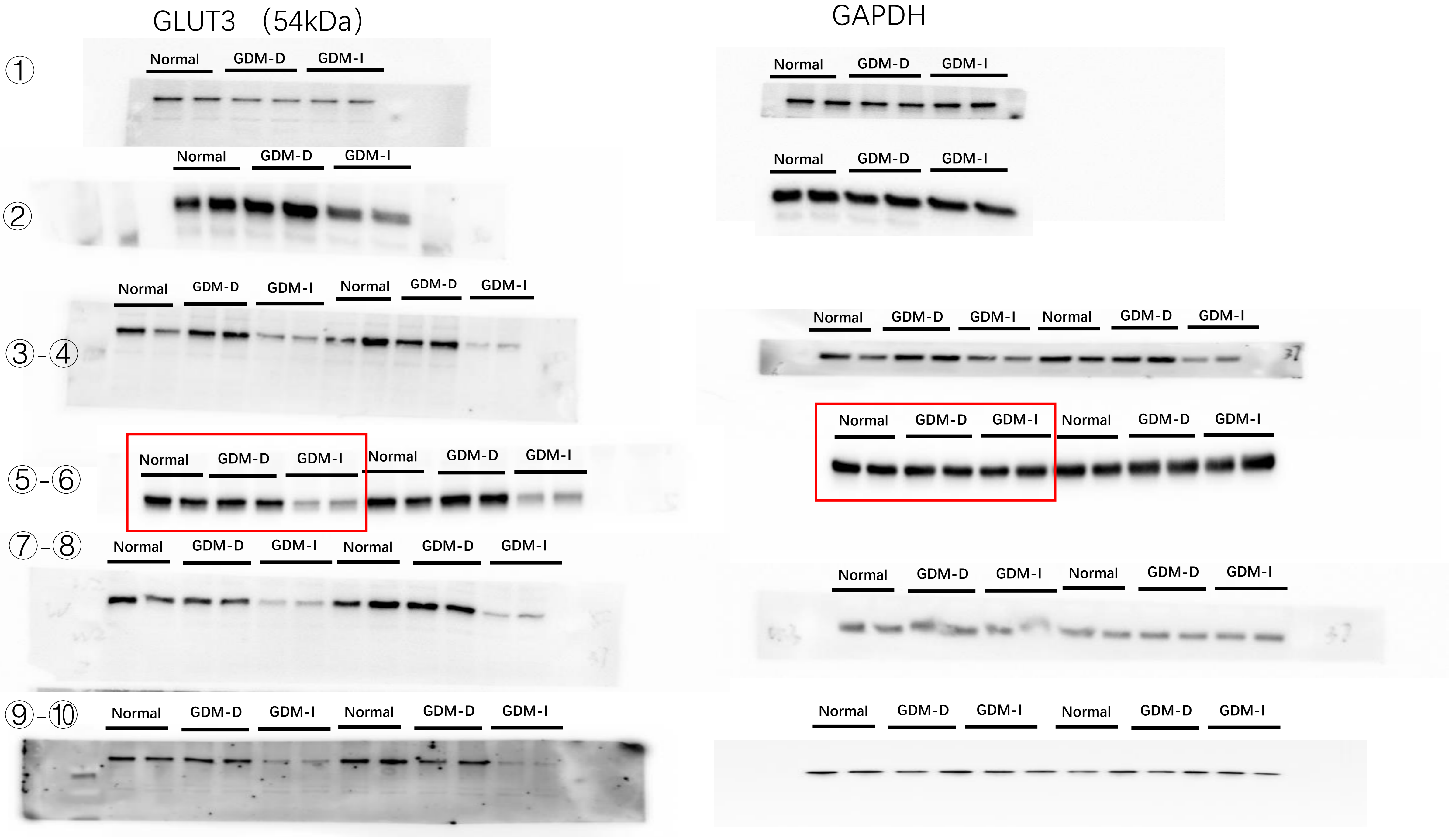
GLUT3 (54kDa)
GAPDH
①
②
③-④
⑤-⑥
⑦-⑧
⑨-⑩
Normal
GDM-D
GDM-I

In the page bellowed, the original blots of cleaved caspase-3 and GAPDH were shown. We analyzed normal, GDM-D, and GDM-I (n=5 per group with duplicate tests), respectively. The levels of cleaved caspase-3 (left side) were normalized to the levels of GAPDH (right side) and the fold change relative to the normal group was calculated in every experiment. Finally, the data were statistics in Fig. 6-A and the blot used in the manuscript is denoted with red lines Fig. 6-C.

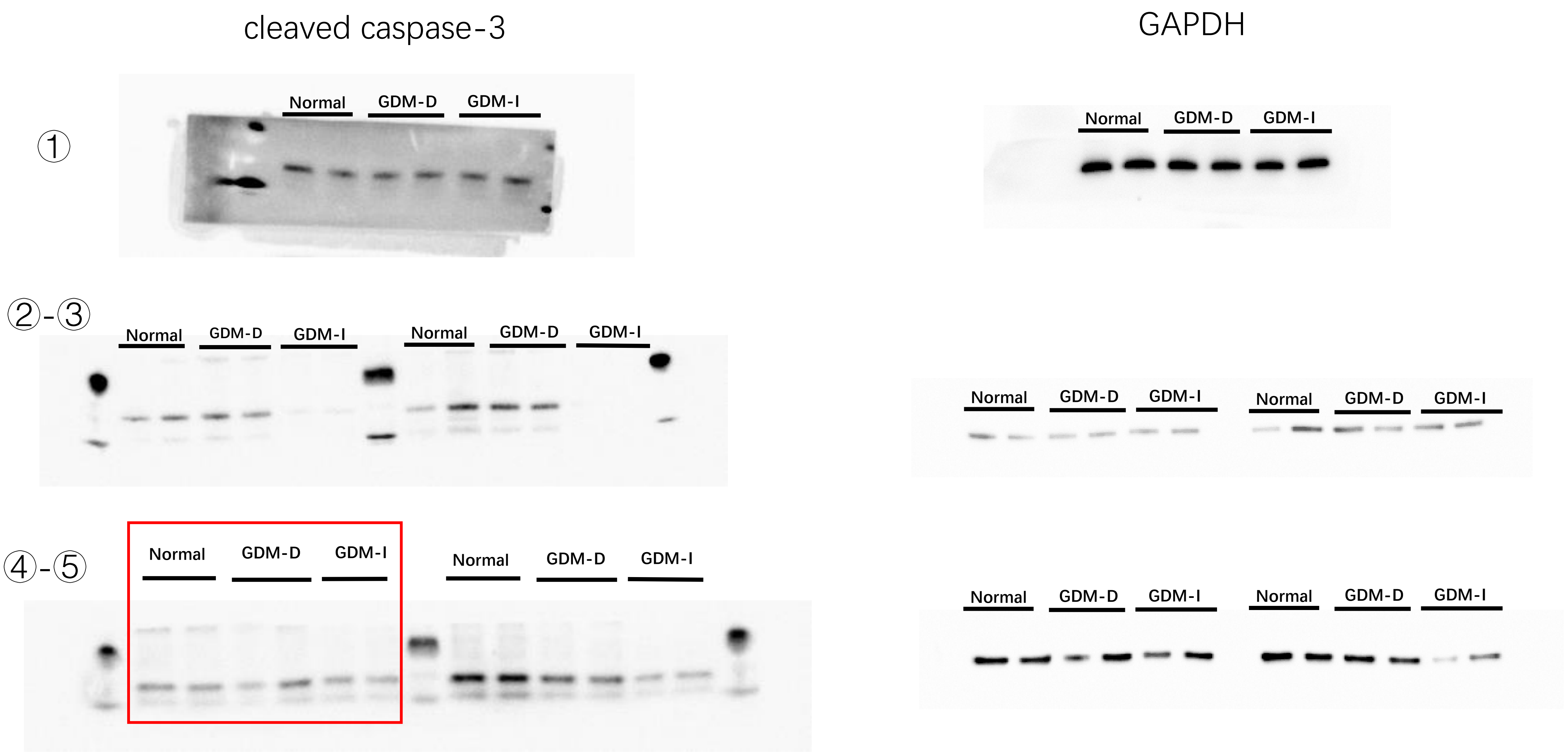
cleaved caspase-3
GAPDH
①
Normal
GDM-D
GDM-I
②-③
Normal
GDM-D
GDM-I
Normal
GDM-D
GDM-I
④-⑤
Normal
GDM-D
GDM-I
Normal
GDM-D
GDM-I

In the page bellowed, the original blots of cleaved PARP and GAPDH were shown. We analyzed normal, GDM-D, and GDM-I (n=9 per group with duplicate tests), respectively. The levels of cleaved PARP (left side) were normalized to the levels of GAPDH (right side) and the fold change relative to the normal group was calculated in every experiment. Finally, the data were statistics in Fig. 6-A and the blot used in the manuscript is denoted with red lines.

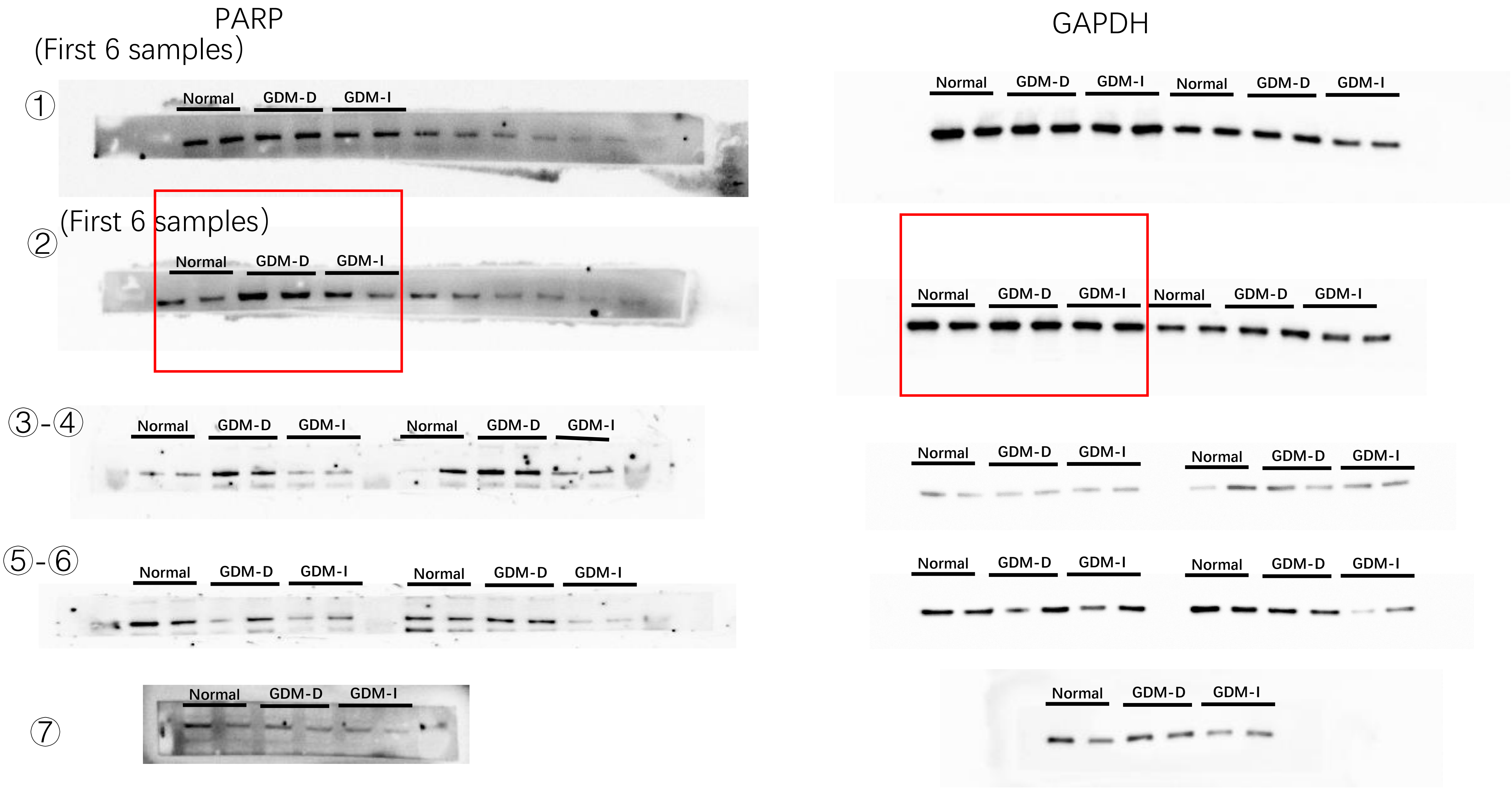

PARP
(First 6 samples)
GAPDH
①
Normal
GDM-D
GDM-I
Normal
GDM-D
GDM-I
Normal
GDM-D
GDM-I
(First 6 samples)
②
Normal
GDM-D
GDM-I
Normal
GDM-D
GDM-I
Normal
GDM-D
GDM-I
③-④
Normal
GDM-D
GDM-I
Normal
GDM-D
GDM-I
Normal
GDM-D
GDM-I
Normal
GDM-D
GDM-I
⑤-⑥
Normal
GDM-D
GDM-I
Normal
GDM-D
GDM-I
Normal
GDM-D
GDM-I
Normal
GDM-D
GDM-I
⑦
Normal
GDM-D
GDM-I
Normal
GDM-D
GDM-I

In the page bellowed, the original blots of phosphate AMPK, total AMPK and GAPDH were shown. We analyzed normal (n=4), GDM-D (n=4), and GDM-I (n=10) per group with duplicate tests), respectively. The levels of both phosphate AMPK and total AMPK were normalized to the levels of GAPDH (right side) and furthermore, the levels of phosphate AMPK (left side) were normalized to the levels of total AMPK (middle). Finally, the data were statistics in Fig. 7-B and the blot used in the manuscript is denoted with red lines.

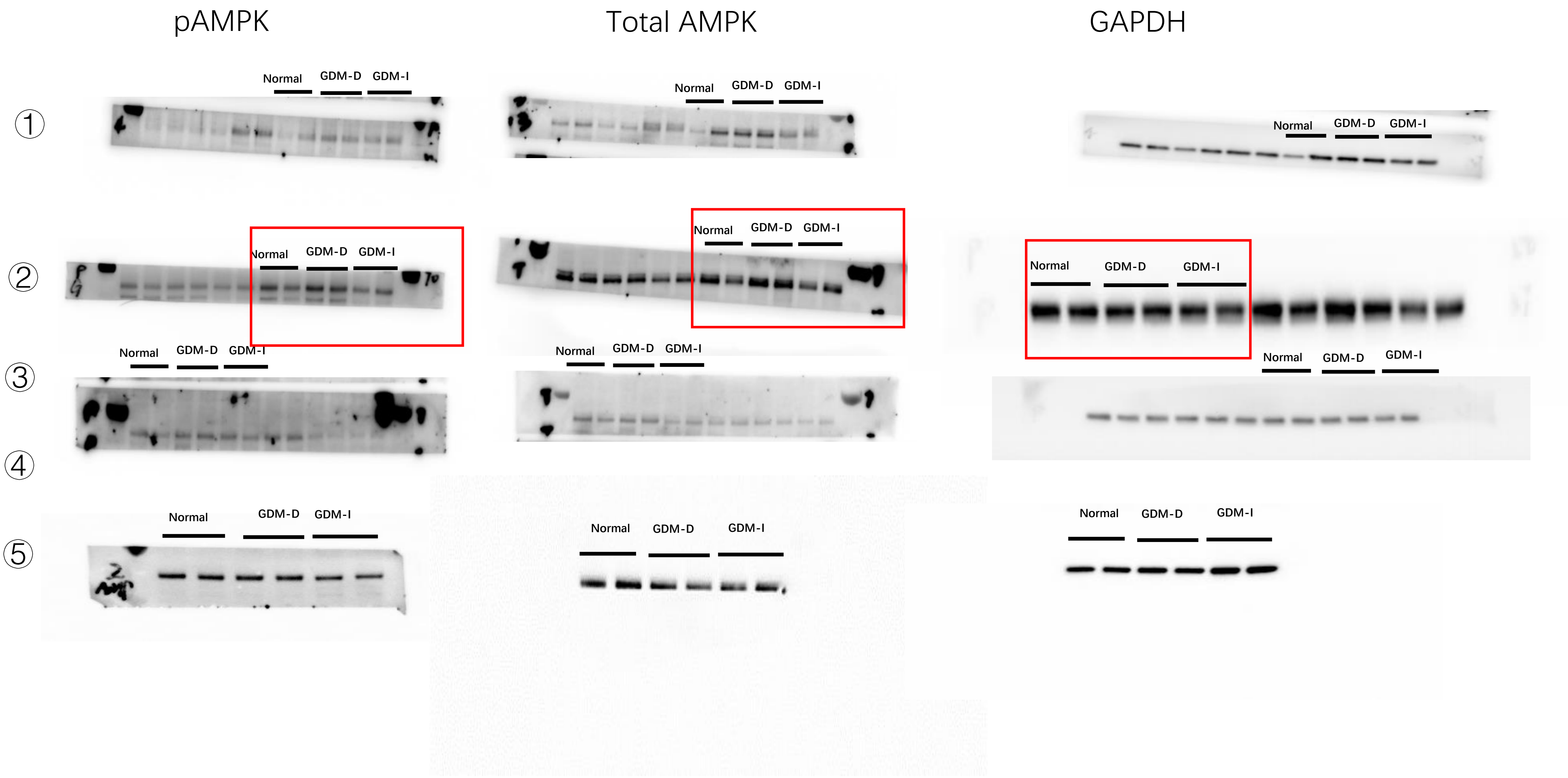

pAMPK
Total AMPK
GAPDH
①
②
③
④
⑤
Normal
GDM-D
GDM-I

In the page bellowed, the original blots of phosphate 4EBP1, total 4EBP1 and GAPDH were shown. We analyzed normal, GDM-D, and GDM-I (n=8 per group with duplicate tests), respectively. The levels of both phosphate 4EBP1 and total 4EBP1 were normalized to the levels of GAPDH (right side) and furthermore, the levels of phosphate 4EBP1 (left side) were normalized to the levels of total 4EBP1 (middle). The fold change relative to the normal group was calculated in every experiment. Finally, the data were statistics in Fig. 7-D and the blot used in the manuscript is denoted with red lines.

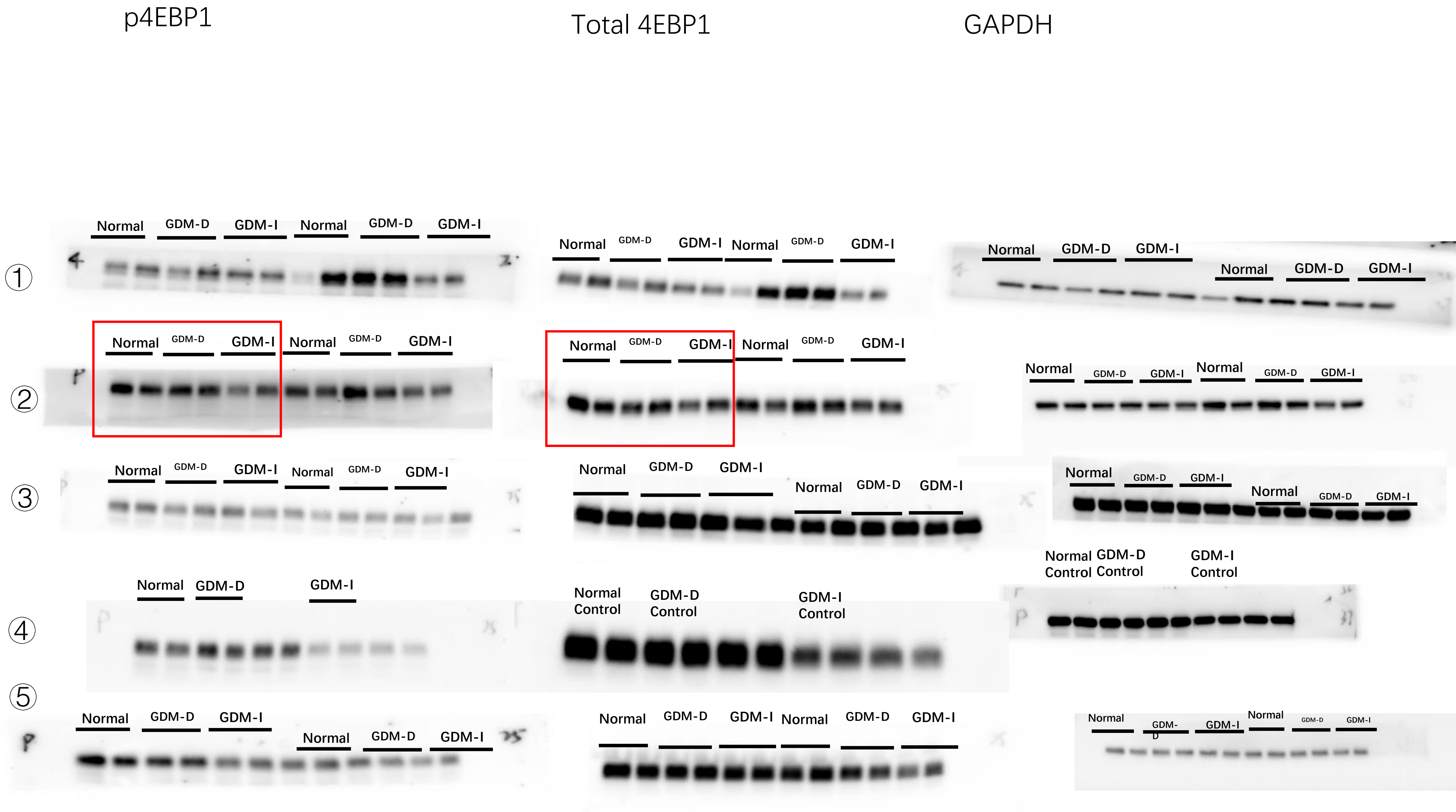
p4EBP1
Total 4EBP1
GAPDH
①
②
③
④
⑤
Normal
GDM-D
GDM-I
Normal Control
GDM-D Control
GDM-I Control

In the page bellowed, the original blots of phosphate mTOR, total mTOR and GAPDH were shown. We analyzed normal, GDM-D, and GDM-I (n=8 per group with duplicate tests), respectively. The levels of both phosphate mTOR and total mTOR were normalized to the levels of GAPDH (right side) and furthermore, the levels of phosphate mTOR (left side) were normalized to the levels of total mTOR (middle). The fold change relative to the normal group was calculated in every experiment. Finally, the data were statistics in Fig. 7-C and the blot used in the manuscript is denoted with red lines.

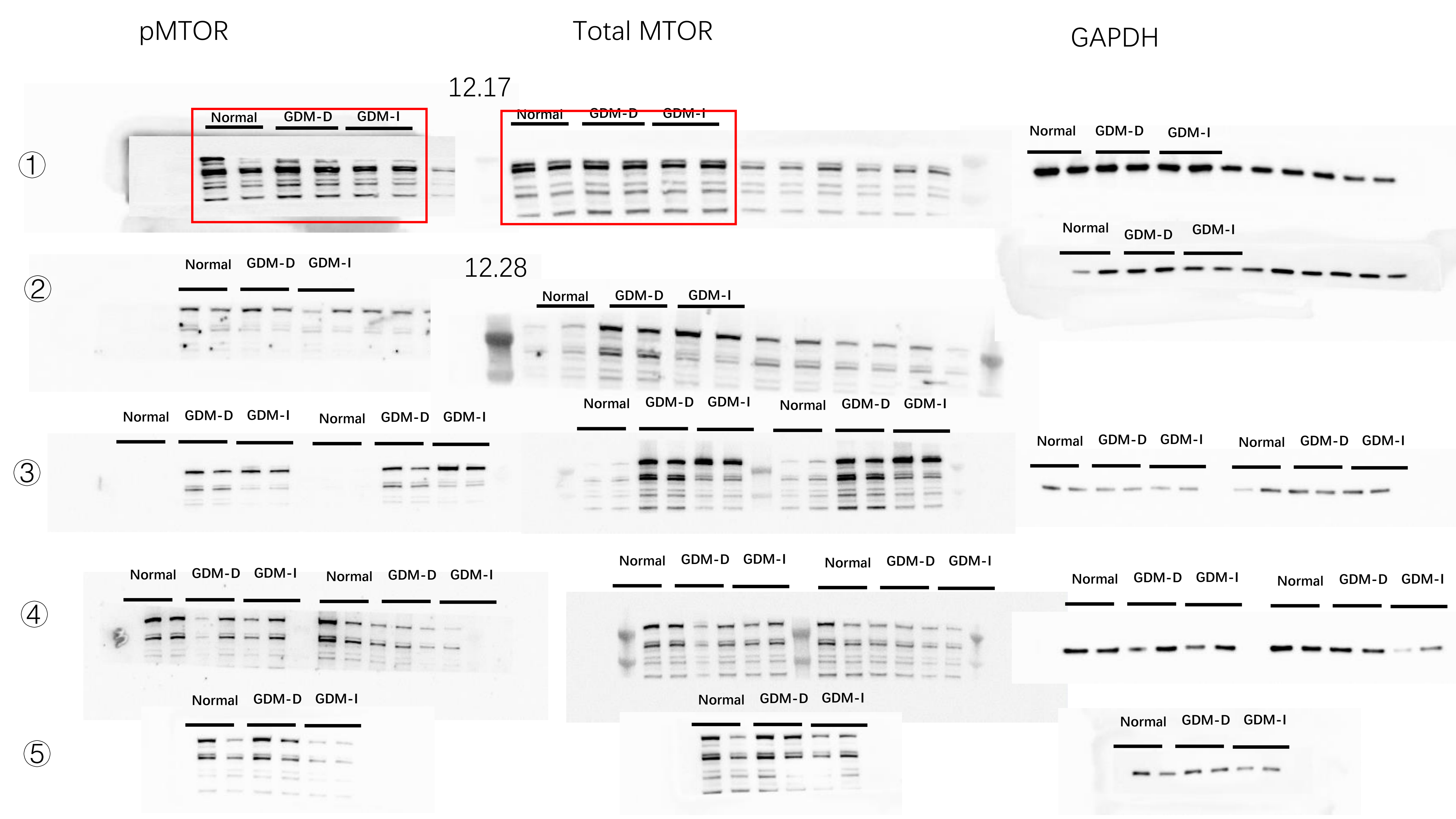
pMTOR
Total MTOR
GAPDH
12.17
12.28
①
②
③
④
⑤
Normal
GDM-D
GDM-I

In the page bellowed, the original blots of phosphate AKT, total AKT and GAPDH were shown. We analyzed normal, GDM-D, and GDM-I (n=8 per group with duplicate tests), respectively. The levels of both phosphate AKT and total AKT were normalized to the levels of GAPDH (right side) and furthermore, the levels of phosphate AKT (left side) were normalized to the levels of total AKT (middle).. The fold change relative to the normal group was calculated in every experiment. Finally, the data were statistics in Fig. 7-A and the blot used in the manuscript is denoted with red lines.

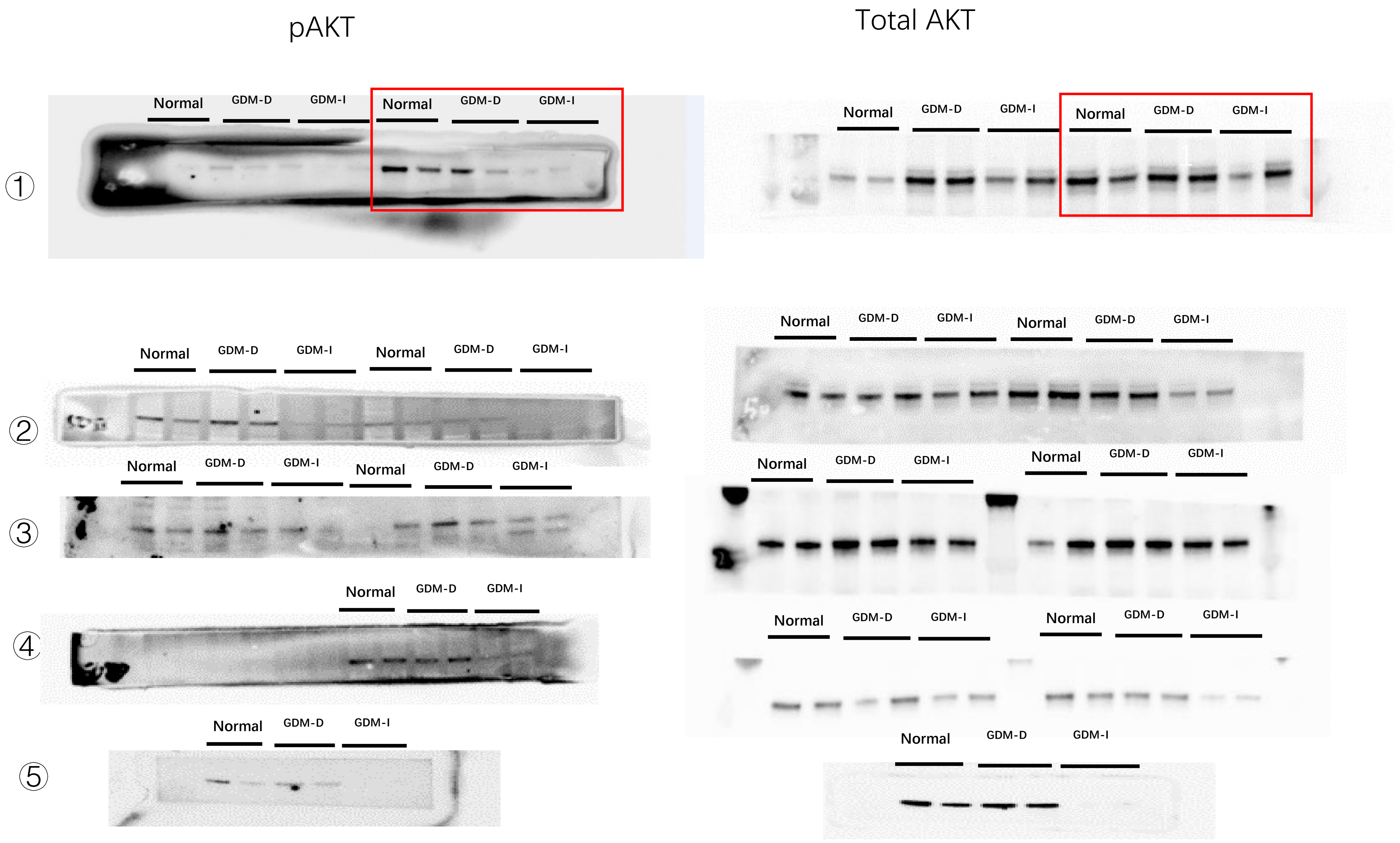
pAKT
Total AKT
①
②
③
④
⑤
Normal
GDM-D
GDM-I

GAPDH

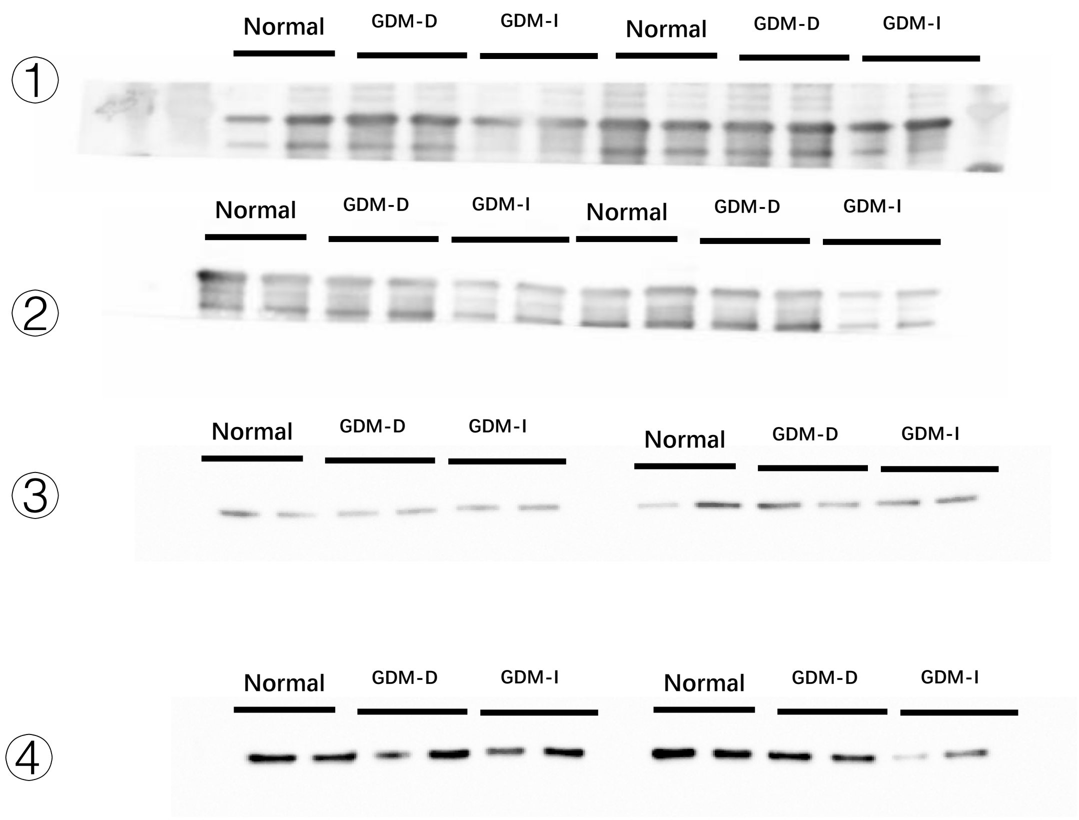

①
Normal GDM-D GDM-I Normal GDM-D GDM-I
②
Normal GDM-D GDM-I Normal GDM-D GDM-I
③
Normal GDM-D GDM-I Normal GDM-D GDM-I
④
Normal GDM-D GDM-I Normal GDM-D GDM-I
